# The Complex Phosphorylation Patterns That Regulate the Activity of Hsp70 and Its Cochaperones

**DOI:** 10.3390/ijms20174122

**Published:** 2019-08-23

**Authors:** Lorea Velasco, Leire Dublang, Fernando Moro, Arturo Muga

**Affiliations:** Biofisika Institute (UPV/EHU, CSIC) and Department of Biochemistry and Molecular Biology, Faculty of Science and Technology, University of the Basque Country (UPV/EHU), Barrio Sarriena s/n, 48940 Leioa, Spain

**Keywords:** chaperones, post-translational modification, phosphorylation, human disaggregase, Hsp40, Hsp70, Hsp110

## Abstract

Proteins must fold into their native structure and maintain it during their lifespan to display the desired activity. To ensure proper folding and stability, and avoid generation of misfolded conformations that can be potentially cytotoxic, cells synthesize a wide variety of molecular chaperones that assist folding of other proteins and avoid their aggregation, which unfortunately is unavoidable under acute stress conditions. A protein machinery in metazoa, composed of representatives of the Hsp70, Hsp40, and Hsp110 chaperone families, can reactivate protein aggregates. We revised herein the phosphorylation sites found so far in members of these chaperone families and the functional consequences associated with some of them. We also discuss how phosphorylation might regulate the chaperone activity and the interaction of human Hsp70 with its accessory and client proteins. Finally, we present the information that would be necessary to decrypt the effect that post-translational modifications, and especially phosphorylation, could have on the biological activity of the Hsp70 system, known as the “chaperone code”.

## 1. Introduction

The amino acid sequence does not dictate the final chemical composition of proteins, as many of them undergo post-translational modifications (PTMs) to form the mature polypeptides that define the cellular proteome [1]. PTMs constitute a main route used by cells to expand and diversify the proteomes way beyond their genomes predict. There are different protein PTMs that include enablers of location, function, and signaling, and markers of stability and degradation. They occur on amino acid side chains or at the protein C- or N-termini, and they extend the chemical properties of the 20 standard amino acids by modifying existing functional groups or introducing new ones (reviewed in [2]). They are found in both prokaryotes and eukaryotes, although being more abundant, frequent and diverse in the latter. About 5% of the genes in eukaryotic DNA code enzymes dedicated to carry out the covalent modification of proteins.

Phosphorylation is the most studied post-translational protein modification, allowing for simple and reversible regulation of protein function. Around 30% of human proteins are phosphorylated during their lifetime [3]. Protein phosphorylation can trigger different and biologically important effects, such as induction of structural changes, protein labeling for cellular translocation and regulation of protein–protein interactions [3]. The phosphorylation process is governed by a finely-tuned interplay of kinases and phosphatases, and can be adjusted in a tissue-specific manner [4]. Here we review PTMs affecting molecular chaperones, a special class of proteins dedicated to promoting and keeping the correct folding of the cellular proteome and solubilize and reactivate protein aggregates.

## 2. Protein Folding and Molecular Chaperones

Proteins are one of the most important and complex components of the cells. The majority of the biochemical processes occurring within a cell rely on proteins acting as catalyzers of complex chemical reactions between biomolecules, transporters, cellular structural scaffolds, etc. The biogenesis of a protein depends on a key process called protein folding by which polypeptide chains acquire a defined three-dimensional structure, usually known as the native state, which features thermodynamic stability and biological activity. Proteins fold using funneled energy landscapes in which the conformational space that the polypeptide has to sample gets restricted towards the native state due to the hydrophobic chain collapse and the increasing number of native interactions [5,6,7]. Many studies have shown that proteins that have been unfolded in vitro can refold spontaneously into their native states in dilute solutions at low temperatures in the absence of any other component, demonstrating that the information required to attain the native state is contained in the primary structure of the polypeptide [8]. However, proteins within cells encounter conditions that are far from the ideal in vitro situation. First, newly synthesized polypeptides have to cope with a highly crowded cellular milieu with a protein concentration of 300–400 g·L^–1^ [9], that modifies folding landscapes and favors off-pathway intermolecular interactions between hydrophobic segments that lead to protein aggregation [10,11]. Second, living organisms can also suffer mutations, different types of environmental stress and aging that hinder folding of polypeptides after ribosomal synthesis and can induce unfolding or misfolding of existing proteins. Finally, these issues are aggravated by the structural flexibility that proteins require to function properly [5,12], making them marginally stable and susceptible to easily unfold exposing aggregation-prone hydrophobic sequences to the aqueous medium. A hallmark of protein folding failure is the formation of aggregates that cells accumulate in specific subcellular compartments as a defense mechanism, sequestering potentially harmful unfolded polypeptides [13,14,15]. Protein aggregates can also be secreted to the extracellular medium by a mechanism not fully understood [16,17]. The risk that protein aggregates represent for cells and living organisms is manifested by their direct relation with human diseases, especially evident in neurodegenerative pathologies characterized by the appearance of amyloid fibers [18].

To compensate the difficulties menacing correct protein folding in vivo, cells have evolved a complex system devoted to facilitate proteins in acquiring and maintaining their native conformations. This protein quality control (PQC) network, also called proteostasis network, comprises molecular chaperones, the components of proteasomal degradation and the autophagy system [19]. Molecular chaperones form coordinated networks to promote de novo folding of polypeptides, rescue misfolded proteins and prevent aggregation of unfolded polypeptides [20]. However, acute and prolonged stress can overcome the buffering effect of the proteostasis network, resulting in the increase of unfolded protein concentration, which in turn derives in aggregation. To ensure survivability, the proteostasis system can resolve cytotoxic protein aggregates by either reactivating their protein components or directing them to proteolytic recycling [21,22,23]. Here we focus on the impact that phosphorylation of members of the Hsp70, Hsp40, and Hsp110 chaperone families might have on their structure and biological activity.

## 3. Molecular Chaperones Involved in Protein Folding and Protein Aggregate Reactivation

**Figure 1:**
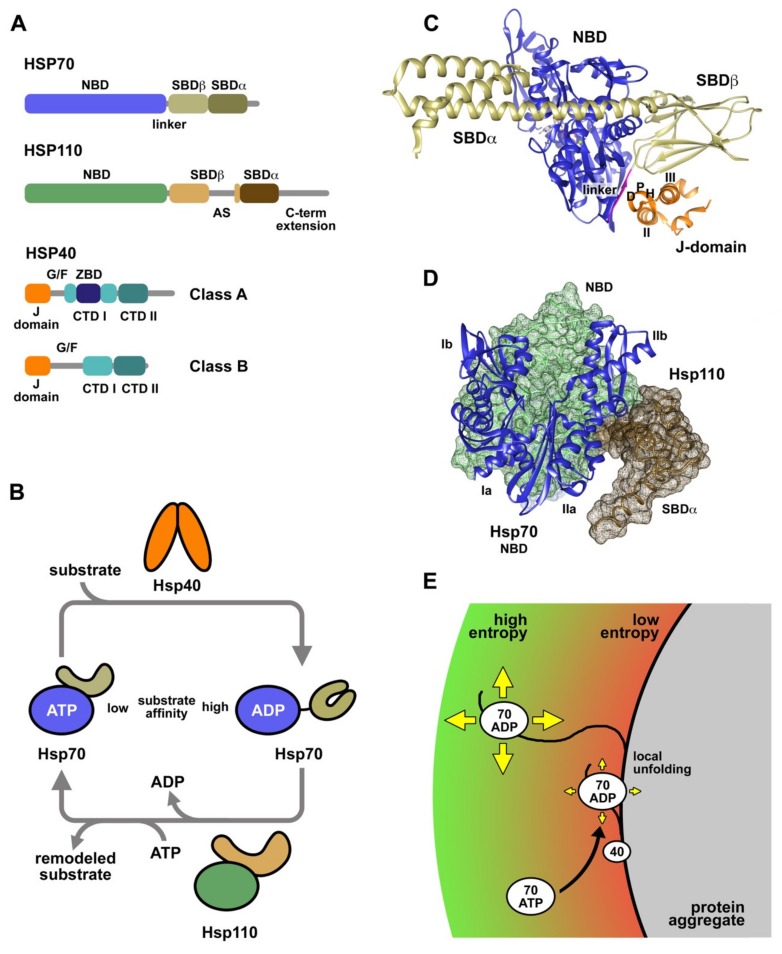
Human chaperones involved in proteostasis and reactivation of protein aggregates. (**A**) Schematic representation of the domain organization of the main proteins comprising the human disaggregase: Hsp70, Hsp110, and Hsp40 (Classes A and B). Proteins and domains are drawn on scale according to the length of their amino acid sequences. (**B**) ATPase and conformational cycle of Hsp70 essential to the chaperone activity. Substrates enter the cycle by binding to Hsp40 and then, they are transferred to the ATP-bound state of Hsp70. Both, the Hsp40 cochaperone and the substrate stimulate ATP hydrolysis, closing the SBD and trapping the substrate in the ADP-state. Hsp110 interacts with the NBD of the chaperone and promotes exchange of ADP by ATP, which triggers the dissociation of the Hsp70-client complex and thus, the release of the substrate. (**C**) Ribbon representation of full-length *E. coli* Hsp70 (DnaK) in complex with the J-domain of DnaJ (PDB ID:5NRO; [31]) using UCSF Chimera [32]. (**D**) Structure of the human Hsp70 NBD in complex with *Saccharomyces cerevisiae* Hsp110 (PDB ID:3D2F) [33]. (**E**) Entropic pulling model proposed to act during protein aggregate solubilization by the Hsp70-system. Hsp40 recruits Hsp70 to the aggregate surface, which results in a reduction of its entropy. Local unfolding of the bound polypeptide might allow the movement of the Hsp70-client complex away from the aggregate surface, incrementing the degrees of freedom of the molecules. This would generate a favorable free energy change (ΔG < 0) due to the local entropy increase.

Hsp70 chaperones participate in a broad range of biological processes, as they prevent protein aggregation, promote the refolding of misfolded denatured proteins, reactivate protein aggregates and collaborate with cellular degradation machineries to clear aberrant proteins and protein aggregates. Thus, Hsp70s protect cells against the detrimental effects of proteotoxic stresses, pathophysiological conditions and ageing that cause protein homeostasis imbalance (review in [24]). The human genome contains at least 13 genes codifying Hsp70 proteins, 6 of which are translated as “canonical” Hsp70s in the cytosol and nucleus (HSPA1A/B, HSPA1L, HSPA2, HSPA6, and HSPA8). HspA8, also termed Hsc70, and HspA1A, represent the major non-inducible and stress-inducible Hsp70s in the cytosol, respectively, and have been found to display significant disaggregase activity on amorphous aggregates and amyloid fibers in vitro [25,26,27]. Different isoforms of Hsp70s are distinguished by their ability to interact with specific Hsp40s and nucleotide exchange factors (NEFs), to recognize specific substrates, to undergo allosteric regulation, and to adapt to the conditions of particular cellular compartments [28,29]. The domain architecture of Hsp70, recently reviewed in [24], consists of N- and C-terminal domains connected by a flexible and highly conserved hydrophobic linker (Figure 1A), essential for allosteric inter-domain communication [30]. The 45 KDa N-terminal domain (NBD) contains a nucleotide binding site with high affinity for ATP and ADP, and low intrinsic hydrolase activity. The NBD has an actin-like configuration with two lobes (I and II) that form a deep cleft where the nucleotide is bound. The C-terminal substrate binding domain (SBD) is able to bind extended polypeptides rich in aliphatic residues and is subdivided in a β-sandwich subdomain (SBDβ) that holds the binding site, an α-helical lid subdomain (SBDα) that locks the substrate, and a C-terminal intrinsically disordered segment. Hsp70 activity requires a sophisticated allosteric coupling between the NBD and SBD: ATP binding induces the release of the bound peptide, and substrate binding stimulates ATP hydrolysis.

Hsp70s can be considered molecular machines that modulate the conformation of their substrate proteins by interacting with short hydrophobic segments exposed to the solvent. The conformational remodeling of the substrates occurs in successive rounds of binding and release, coupled to the nucleotide-dependent conformational cycle of Hsp70 (Figure 1B). In the ATP conformation, the SBD is docked onto the NBD, displaying a low affinity for substrates due to their fast association and dissociation kinetics [34,35]. Hsp40 cochaperones and the substrate synergistically stimulate ATP hydrolysis, which induces a large structural rearrangement that includes NDB and SBD disengagement and lid closure on the substrate, thus enhancing the stability of the chaperone-client complex [36]. Nucleotide exchange factors, as human Hsp110 proteins, promote ADP/ATP exchange in Hsp70, restarting the cycle and releasing the substrate to the medium. Using this mechanism, DnaK, the main bacterial Hsp70, functions as an unfoldase, allowing a misfolded mutant of luciferase to reach the native state [37].

Hsp40s, also called J proteins, constitute a less conserved and larger chaperone family, compared to Hsp70s, with at least 41 representatives in humans [28,38]. All members of the Hsp40 family share a conserved domain of approximately 70 residues, called J-domain, usually located in the N-terminus, which is essential to stimulate the ATPase activity of Hsp70s [39,40]. Hsp40s are classified in three classes depending on their domain organization and composition (Figure 1A): i) Class A (or Type I) contains a J-domain followed by a G/F-rich region, a zinc binding domain (ZBD), and a C-terminal domain (CTDI and II) that ends in a dimerization motif and has the ability to interact with peptides rich in aromatic residues [41]; ii) Class B (or Type II) proteins have a similar domain composition and organization but lack the ZBD; iii) and Class C (or Type III) is a highly diverse group with specific functions that only shares with the other groups the presence of the J-domain. The diversity of the Hsp40 family is associated with its ability to drive the function of Hsp70s to a plethora of specific processes and subcellular localizations [28]. Class A and B Hsp40s are ATP-independent cochaperones of Hsp70s that assist in the quality control of the proteome. Human DnaJB1, the main cytosolic heat shock inducible class B Hsp40, displays disaggregase activity in cooperation with Hsc70 [25,26,27]. Aggregate reactivation by Hsc70 is enhanced when DnaJB1 is combined with DnaJA2, a class A Hsp40, due to the formation of transient complexes between both J proteins that might tether higher order Hsc70 supercomplexes [42].

Hsp40s exhibit holdase activity, due to their ability to bind substrates, preventing their aggregation and most likely modifying their conformation [43,44,45]. Small peptides bind in a hydrophobic pocket located in the CTD [41], whereas recognition and stable interaction with client proteins also involve the zinc binding and the G/F domains [43,46,47]. Hsp40s transfer substrates to Hsp70 by a mechanism poorly understood, concomitantly stimulating the ATPase activity of the chaperone and trapping of the substrate [48]. The structure and the sequence of the J-domain are highly conserved in the Hsp40 family. This domain is formed by four α-helices (I-IV) folded around a well-defined hydrophobic core [49,50]. The recently published structure of the complex between *Escherichia coli* DnaK and the J-domain of DnaJ reveals the details of the interaction (Figure 1C) [31]: (1) the common HPD motif contacts residues at the linker, NBD and SBD of DnaK; (2) helix II establishes interactions with the linker and NBD; (3) and helix III interacts with the SBD. Using this tripartite interaction interface, the J-domain can sense the occupancy of the substrate binding site and concomitantly stimulate ATP hydrolysis. It is important to mention that all the essential residues engaged in these contacts between the *E. coli* proteins are conserved from bacteria to humans [51]. Other regions involved in the interaction of Hsp40 with Hsp70 chaperones are the CTD of Hsp40s and the SBD and conserved C-terminal EEVD motif of cytosolic Hsp70s [52,53,54,55]. The EEVD motif of Hsp70 is also involved in the interaction of Hsp70 with specific cofactors, such as HOP, which uses its three tetratricopeptide repeat (TPR) domains to bind the C-terminal EEVD motifs of Hsp70 and Hsp90, thus facilitating substrate handover between these foldases [56]. On the degradation pathway, CHIP also associates with this motif of Hsp70 through its TPR domain, binding at the same time the enzymes necessary to ubiquitylate the substrates that will be target for degradation [57,58].

Hsp110 chaperones are the remaining components in metazoan cells required for, among other functions, an efficient protein aggregate reactivation in vitro and in vivo [25,26,59]. The human genome contains three isoforms belonging to this family: Hsp105 (HSPH1), Apg2 (HSPH2), and Apg1 (HSPH3), all of which support in vitro reactivation of protein aggregates by HspA8 and DnaJB1 [26]. Hsp110 proteins are closely related to Hsp70s and share a similar domain organization, e.g., they contain the aforementioned NBD and SBD [60] connected by an amphipathic linker instead of the conserved hydrophobic linker of Hsp70s (Figure 1A). Hsp110s are larger proteins due to the insertion of a proline-rich acidic subdomain (AS) in the β-sandwich of the SBD, and an extension of the intrinsically disordered C-terminal segment [21]. The AS is involved in the interaction with Hsp70 and in the regulation of its ATPase/conformational cycle [61], and both the AS and the C-terminal domain participate in the correct intracellular localization of the isoforms α and β of Hsp105 [62,63]. Although Hsp110s exhibit ATPase activity, they lack the Hsp70-like allosteric docking/undocking of NBD and SBD coupled to ATP binding and hydrolysis, displaying limited conformational changes [64,65,66]. Proteins of the Hsp110 family act as holdases, binding and protecting unstable substrate proteins from aggregation [67,68], and regulate the ATPase and conformational cycle of Hsp70s functioning as nucleotide exchange factors [69,70]. They promote fast nucleotide exchange rates, clamping lobe IIb of Hsp70 NBD and inducing a sideways rotation that opens the nucleotide binding cleft and reduces the chaperone affinity for ADP. Hsp110 and Hsp70 form stable complexes stabilized by an extensive interaction interface formed by the NBDs of both proteins and the C-terminal SBDα of Hsp110 (Figure 1D) [33,71].

## 4. Interaction of Chaperones with Substrate Proteins and Protein Aggregates

Molecular chaperones play a pivotal role in maintaining protein homeostasis in the cell by modulating protein conformational states and regulating the transition from the native protein conformation to the aggregated or amyloid states [72,73,74]. Among them, Hsp70s have an essential role in protein folding, disaggregation, and degradation. The recently proposed model for Hsp70 functioning as a “multiple socket” postulates that Hsp70 provides a physical platform for the binding of client proteins, other chaperones, and cochaperones. The final destiny of the client protein is regulated by the combination of Hsp70 interactions that occur in different cellular contexts. In collaboration with Hsp90 and several cofactors, as Hsp40s and Hop, coordinates the folding and maturation of key regulatory client proteins. In complex with CHIP and specific NEFs, directs the substrate to proteasomal degradation, whereas the interaction with specific Hsp40s and Hsp110-type NEFs engages the chaperone in the reactivation of protein aggregates [75]. It is worth mentioning that the interaction of chaperones with native protein conformations regulates important biological activities, and that this interplay is sensitive to their phosphorylation status (see below). A detailed list of the chaperone substrates related to neurodegenerative pathologies can be found in [76]. Chaperones follow different strategies to fight protein misfolding and aggregation. First, they transiently interact with aggregation-prone regions of misfolded monomeric proteins or intrinsically disordered proteins, inhibiting their initial oligomerization into seeding competent aggregates and facilitating the folding into their native state once the proteotoxic stress ceases. This initial step is essential for aggregation to occur, and therefore its inhibition could solve aggregation-associated diseases. Canonical Hsp40s, sHSPs, Hsp70, and Hsp110 follow this strategy, whereas noncanonical Hsp40s prevent primary nucleation by stabilizing oligomeric states before they convert into aggregation seeds (review in [76]). Chaperones can also neutralize the toxic protein oligomers by forming higher-order, mixed complexes that hamper their unspecific interaction with other cellular proteins, thus reducing their toxicity [77,78]. They can also reverse protein aggregation, either passively, binding monomers that dissociate from aggregates or fibrils, or actively, accelerating depolymerization and fragmentation of fibrils. Amyloid fibrils of αsyn [27,79] and Htt Exon1 [80] can be disaggregated by the cooperative action of human HSPA8 (Hsc70), DNAJB1, and NEFs of the Hsp110 family.

Solubilization of polypeptide chains depends upon the interaction of the chaperones with the aggregate, which is most likely the rate limiting step of the reactivation reaction, as protein aggregate solubilization correlates with refolding of the extracted unfolded molecules [61,81,82]. Similar to the bacterial system, the initial binding of DnaJB1 to the aggregate surface efficiently recruits Hsc70 [61,83]. Apg2 further increases binding of Hsc70 in agreement with the refolding stimulation provided by Hsp110 [25,26,61]. In contrast to Hsc70, Apg2 interacts poorly with protein aggregates, being more effective in recruiting Hsc70 and promoting aggregate reactivation at substoichiometric concentrations [26,61]. These findings indicate that Apg2 possibly plays a catalytic role in the solubilization of protein aggregates compatible with its nucleotide exchange role, and suggest that the human (metazoan) disaggregase core is built by Hsc70 and Hsp40 proteins. Different models have been proposed to illustrate how Hsp70 chaperones exert force on polypeptides: the traditional power stroke and molecular ratchet, and the currently most accepted, entropic pulling model (Figure 1E) [84,85]. This model considers the thermodynamic behavior of molecules in constrained spaces. Hsp70 molecules bound to aggregated polypeptide chains experience a considerable reduction of their degrees of freedom and, therefore, of their entropy, since they are apposed against the aggregate surface. Binding of Hsp70 might induce localized partial unfolding of the polypeptide favoring movement of the chaperone away from the surface, along with the bound substrate. This would result in an immediate increase in entropy, which could be translated into a favorable free energy change that can be converted into directional force to unravel polypeptides from the aggregate.

## 5. Regulation of Chaperone Activity by Phosphorylation

Recent studies indicate that molecular chaperones undergo PTMs, and that their phosphorylation regulates important cellular processes, such as cell cycle progression, apoptosis, protein degradation, resistance to anticancer therapeutics, and host–pathogen interaction. We focus in this section on the effect that phosphorylation of specific residues of members of the Hsp40, Hsp70, and Hsp110 chaperone families has on their biological function (summarized in Table 1).

### 5.1. Hsp40

**Figure 2:**
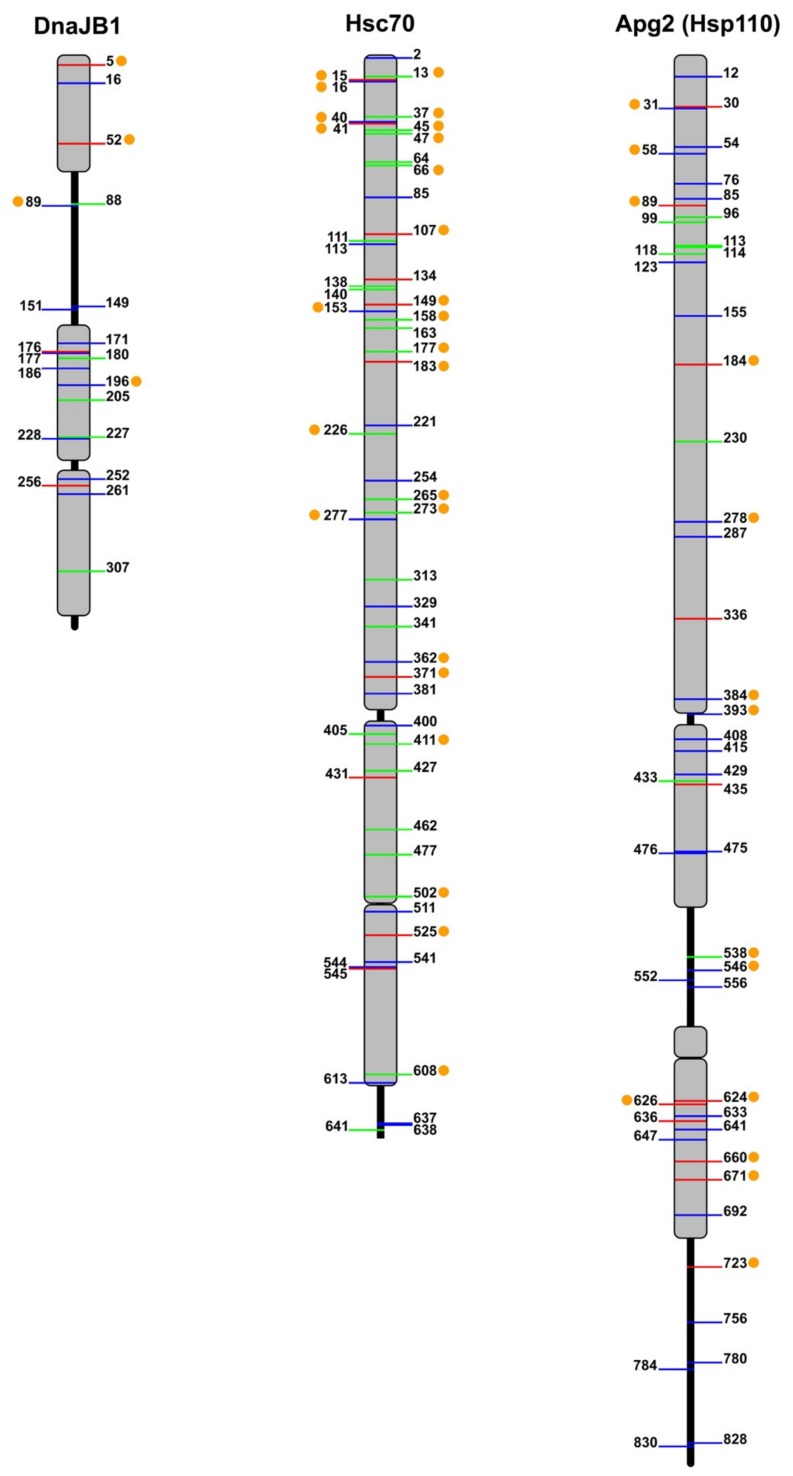
Mapping of phosphosites in representatives of the human Hsp40, Hsp70, and Hsp110 families. Phosphothreonines (green), phosphoserines (blue) and phosphotyrosines (red) listed in PhosphositePlus are shown for DnaJB1, Hsc70 (HspA8), and Apg2 (HspA4 or HspH2). Orange dots represent conserved residues in canonical, cytosolic members of the different chaperone families, which were found phosphorylated in at least two members of the corresponding families.

Heat shock protein 40 (Hsp40) acts as a cochaperone of the Hsp70 family, to promote protein folding, transport, and degradation [100]. The human Hsp40 family contains more than 41 members, some of which can exist as phosphoproteins in the cell (Figure 2; see PhosphoSitePlus -http:// www.Phosphosite.org-) [101]. However, information on the protein kinases and phosphatases responsible for their (de)phosphorylation and the functional relevance of this post-translational modification is scarce. A few examples have succeeded in the identification of the kinases involved in phosphorylation of specific Hsp40 residues.

One of these studies has shown that Hsp40/DnaJB1 is a substrate for mitogen-activated protein kinase 5 (MK5) [86]. MK5 and DnaJB1 form complexes in cells, which are stabilized through interactions between the C-terminal regions of both proteins. This interaction abrogates phosphorylation of DnaJB1 at several residues in vitro, whereas in vivo the chaperone is phosphorylated at Ser-149 or/and Ser-151 and Ser-171. These residues are conserved in DnaJB1 from other species, underscoring the importance of these putative phosphorylation sites. Substitution of these three amino acids by Ala did not completely abolish phosphorylation of the proximal C1 fragment, which encompasses residues 106–175, suggesting that other residues, such as Ser-132, Thr-142, and Thr-165, which are putative phosphorylation sites within this fragment, may also function as MK5 phosphoacceptor sites. Additional phosphorylation sites might also be located at other regions of DnaJB1 (see below), for instance Ser-16 at the J-domain, which is also highly conserved in DnaJB1 from other species, could also be phosphorylated in vitro. Furthermore, the finding that substitution of this residue by non-phosphorylatable Ala did not cancel in vitro phosphorylation of the J-domain by MK5, also suggests that other residues (Thr-8 and Ser-56), might be potential target sites for this kinase.

MK5-dependent phosphorylation of the Hsp70/DnaJB1 mixture stimulates the ATPase activity, suggesting that phosphorylated DnaJB1 may enhance the functional cycle of Hsp70. Although the precise mechanism of action still awaits to be unraveled, phosphorylation of DnaJB1 by MK5 also stimulates repression of the transcriptional activity of HSF1. It seems that the cochaperone does not hamper its binding to DNA, but rather interacts with the trans-activation domain of HSF1, changing its conformation [102].

**Figure 3:**
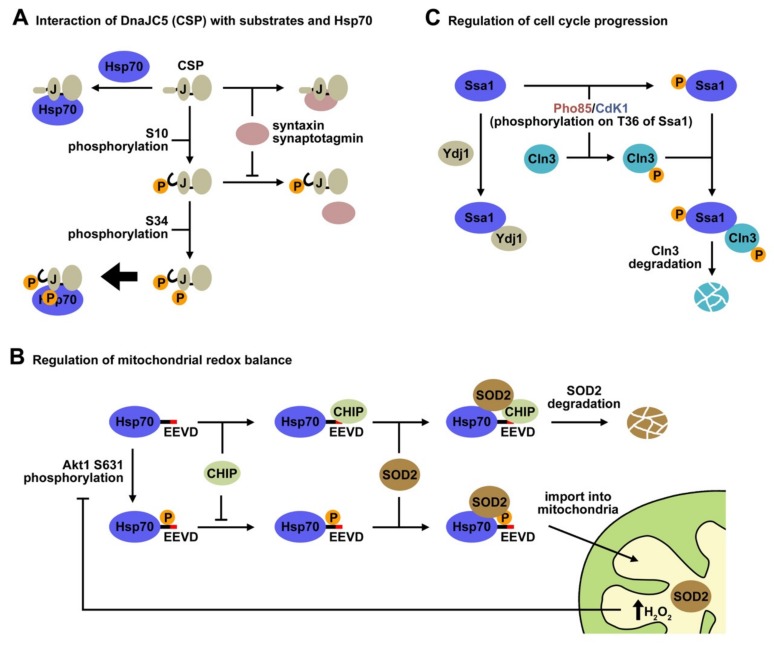
Phosphorylation of members of the Hsp40 and Hsp70 chaperone families regulates important physiological processes. (**A**) Phosphorylation of specific residues of CSP (DnaJC5) controls its interaction with different partners. Posphorylation at Ser10 induces an order-to-disorder transition in the N-terminal domain of CPS that weakens its interaction with syntaxin and synaptotagmin, but does not alter its bindig to Hsp70 [87]. However, double phosphorylation at Ser10 and Ser43 favors its interaction with Hsp70, resulting in a better chaperone activity that supports neuronal cell survival [88]. (**B**) Reversible phosphorylation of Hsp70 at Ser631 plays a key role in the regulation of mitochondrial redox balance. Hsp70 assists SOD2 in its efficient translocation to the mitochondria and also drives SOD2 to degradation in complex with CHIP. Phosphorylation on Ser631 inhibits Hsp70-CHIP complex formation, thus promoting translocation of SOD2 into the mitochondria. The antioxidant activity of SOD2 increases the concentration of H_2_O_2_ within the mitochondria, inducing expression of a phosphatase that deactivates Akt1, the kinase that phosphorylates Hsp70. This results in a lower Hsp70 phosphorylation rate, favoring CHIP binding to Hsp70 and SOD2 degradation [90]. (**C**) Yeast Hsp70 (Ssa1) modulates the cell cycle via its phosphorylation on Thr34, which occurs under nutrient limiting conditions or during the G2/M phase. Phosphorylation of Ssa1 on Thr34 softens its interaction with Ydj1 and promotes chaperone binding to phosphorylated Cln3, which leads to Cln3 degradation and to the next G1 phase [96].

Another example of a member of the Hsp40 protein family that undergoes phosphorylation is the cysteine string protein (CSP), which localizes to neuronal synaptic vesicles. CSP belongs to the class C (DnaJC5) Hsp40 and is highly expressed in all neurons, where it performs a universal neuroprotective function [103], especially at the presynaptic terminal. Loss of function of this protein is related to neurodegeneration in humans and model organisms due to misfolding of client proteins involved in neurotransmission. It binds misfolded proteins, preventing their aggregation, and stimulates the ATPase activity of the 70 kDa heat shock cognate proteins (Hsc70/Hsp70) to regulate protein folding [104]. CSP contains unique domains different from the evolutionarily conserved, characteristic J-domain. The cysteine string domain comprises 13–15 cysteine residues in an approximately 25 amino acid motif, most of which are palmitoylated [105]. This domain is essential for targeting CSP to synaptic vesicles and for neurotransmitter release in vivo. The C-terminal domain displays relatively low sequence conservation among CSP homologs from various species, and its function is poorly understood. Finally, CSPs contain a short N-terminal polypeptide sequence that is phosphorylated in vivo from worms to humans [106,107,108]. Phosphorylation of mammalian CSP on Ser10 inhibits binding to syntaxin and synaptotagmin, but not to Hsc70 [109], and modulates cellular exocytosis release kinetics [110] (Figure 3A). A recent NMR study has shed light on the Ser-10 phosphorylation-dependent conformational change that explains the regulation of CSP activity by this PTM. The solution structure of the serine10-phosphorylated, N-terminal region of CSP (pCSP1-100) reveals an order-to-disorder transition, which results in a more compact overall structure of pCSP1-100, and in a significant modification of its surface charge distribution [87]. The conformational phospho-switch reported in this study provides a structural basis for the previously established effects of Ser10 phosphorylation on CSP function. This structural change destabilizes and reduces the accessibility of the N-terminal α1 helix, which might explain weakening of specific protein–protein interactions involving this region, such as complex formation of CSP with syntaxin and synaptotagmin. Interestingly, the overall structure of the J-domain and the accessibility of the HPD motif required for Hsp70 activation are unaffected by Ser10 phosphorylation, as expected from the absence of effect of CSP phosphorylation on its interaction with Hsp70 [107] (Figure 3A).

It is important to note that the Lys58 residue that interacts with phospho-Ser10 in CSP is one of the most highly conserved residues in DnaJ proteins [104], and can be ubiquitinated [111] as the orthologous Lys residues in human DnaJA1 and DnaJB1. The tight interaction of phospho-Ser10 with Lys58 could regulate the accessibility of E3 ligases, thereby antagonizing CSP ubiquitination. This finding put forward an attractive mechanism that may regulate protein conformation through the antagonistic effect of posttranslational modifications. If we consider that 36 of the 41 DnaJ proteins encoded by the human genome are phosphorylated in serine/threonine residues [112], the phosphorylation-induced conformational transition reported for CSP could also apply for the regulation of other DnaJ/Hsp40 chaperones.

Although it seems clear that phosphorylation of CSP at Ser10 does not modify its interaction with Hsc70 [112], a recent work by Shirafuji et al. has demonstrated that double phosphorylation of CSP at Ser10 and Ser34 by protein kinase C (PKC) promotes the interaction between CSP and Hsp70/Hsc70 [88] (Figure 3A). This interaction further enhances their chaperone activity for SNAP25 and eventually supports neuronal cell survival. Therefore, PKC-phosphorylation of human CSP at Ser34 in the helix II of the J-domain is assumed to facilitate complex formation with Hsp70/Hsc70. An alternative explanation is that phosphorylation of both sites triggers the conformational change that further stabilizes the Hsp40-Hsp70 complex. Nevertheless, these findings indicate that phosphorylation of different residues along the polypeptide chain regulates the interaction of CSP with specific partners, depending on whether the phosphorylated regions are involved in complex formation or induce conformational changes that favor the interaction with one of them.

### 5.2. Hsp110

Multiple phosphorylation sites have also been detected in the three isoforms of human Hsp110 (Figure 2 lists phosphosites found in Apg2; see PhosphoSitePlus -http:// www.Phosphosite.org-) [101]. Unfortunately, the effect of only one of these phosphorylation sites on the functional properties of Hsp105α has been characterized [89]. A combination of peptide mapping analysis and the use of several mutants of this protein reveals that Ser509 is phosphorylated by CK2. This residue was also found phosphorylated in mammalian COS-7 cells, although other sites were modified as well. This PTM regulates the association of Hsp110 with Hsc70, affecting mainly to its dissociation from Hsc70 and therefore to the ability of the Hsp70 system to reactivate luciferase aggregates.

### 5.3. Hsp70

Hsp70 is a highly conserved chaperone implicated, as aforementioned, in three main general and essential processes: Protein folding and protein aggregate reactivation, regulation of protein–protein interactions, and degradation of misfolded proteins [24]. The main regulation factors of the Hsp70 activity in cells are HSF1 and the accessory proteins or cochaperones. Recently, phosphorylation has appeared as an additional layer of complexity in the regulation of Hsp70 function [113]. An attractive hypothesis, recently put forward, postulates that specific phosphorylation patterns, similar to those described for the histone-code, may fine-tune Hsp70 activity [114].

The combined use of global and targeted phosphoproteomics has uncovered 54 phosphorylation sites on Hsc70 (Figure 2) (see PhosphoSitePlus -http:// www.Phosphosite.org-). These essential studies, however, do not infer the role of these modifications or their combination in the regulation of chaperone function. When this large number of potential phosphorylation sites are analyzed considering only those that are predicted to be involved in protein–protein interaction or enzyme activity, the list is reduced to 313 phosphosites on Hsp70 isoforms across 11 species [115]. We analyze below the specific functions of Hsp70 that have been associated with phosphorylation of concrete residues.

#### 5.3.1. Regulation of the Mitochondrial Redox Balance

It has been reported that the C terminus of Hsp70 contains phosphorylation sites for kinases such as Casein kinases [94]. Phosphorylation of this chaperone region regulates import of superoxide dismutase-2 (SOD2) into the mitochondria and the redox balance (Figure 3B). SOD2 is a member of the SOD family of antioxidants, and protects cells against mitochondrial oxidative damage [116]. It contains a mitochondrial targeting sequence that drives it across the outer and inner mitochondrial membranes into the mitochondrial matrix, where it binds manganese (Mn2+) [117]. A peptidase cleaves the mitochondrial targeting sequence, yielding a fully active protein [118]. Although the mitochondrial targeting sequence harbors the information essential for SOD2 to navigate the cytoplasm and to translocate into mitochondria, SOD2 requires assistance from Hsp70 to commit the enzyme to mitochondrial translocation pathways [119]. Hsp70 recognizes and binds short hydrophobic sequences on the amino terminus of SOD2, prevents its aggregation and presents it to the translocation machinery in an import-competent conformation [120]. In addition to the folding events, Hsp70 also directs SOD2 to the ubiquitin-proteasome system (UPS) for degradation [121], and therefore regulates the abundance of this protein.

A recent study has demonstrated that phosphorylation/dephosphorylation cycles of Hsp70 control mitochondrial redox balance by modulating CHIP-mediated degradation of SOD2 [90]. Phosphorylation of Hsp70 on Ser631 by Akt1 decreases its affinity for CHIP, and thus promote the import of SOD2. Phosphorylation induces a structural change in the Hsp70 conformation that enhances its ability to refold and transport SOD2 to the mitochondria. An increase in the concentration of mitochondrial H_2_O_2_, a product of the SOD2 antioxidant activity, inhibits further import of SOD2 by inducing the expression of PP2C, a protein phosphatase that deactivates Akt1 kinase and decreases the rate of Hsp70 phosphorylation. These observations have led to a model of SOD2 signaling whereby, following cell stimulation, Hsp70 is phosphorylated and increases SOD2 import. The transient nature of this response is achieved by rapid dephosphorylation of Hsp70, which inhibits SOD2 import and activity. This study strongly suggests that reversible phosphorylation of Hsp70 could be a physiological mechanism for the regulation of processes as important as the mitochondrial redox balance.

#### 5.3.2. Host-Pathogen Interaction

Hsp70 phosphorylation has also been involved in the regulation of host–pathogen interactions. A recent study has shown that *Legionella pneumophilia* (L.p.) targets Hsp70 to reduce host translation [92]. L.p is a model organism for studying host–pathogen interactions, as many key regulatory pathways, including host translation and eukaryotic vesicle transport, can be easily manipulated. To control these host processes, L.p. uses a type IV secretion system to translocate approximately 300 bacterial effector proteins directly into infected host cells [122]. One of this factor, a eukaryotic-like, Ser/Thr effector kinase known as LegK4 [123], phosphorylates Hsp70 at Thr495 in the substrate-binding domain, disrupting its ATPase activity and greatly inhibiting its protein folding capacity. This results in translation inhibition and in an increase in the amount of Hsp70 bound to highly translating polysomes. Phosphorylated Hsp70 might be unable to fold nascent polypeptides correctly and thus, remains associated with the polysomes longer than usual. The ability of LegK4 to inhibit host translation via a single phosphorylation uncovers a role for Hsp70 in protein synthesis and directly links it to the cellular translational machinery. This study also describes a pathogen using a kinase to phosphorylate host Hsp70 during infection.

#### 5.3.3. Regulation of Hsp70 Dimerization by Phosphorylation

Hsp40 mediates complex formation between Hsp70 and client proteins prior to interaction with Hsp90 [24]. The Hsp70/90 system requires a plethora of accessory proteins to provide specificity and regulate its interactions with client proteins [124]. Hsp70 binds extended hydrophobic peptide sequences and acts at an early stage to recognize partially folded client proteins, unlike Hsp90 that is believed to interact with substrates in a near-native conformation. In contrast to Hsp90, Hsp70 is primarily monomeric in solution [125], although dimerization has also been reported for DnaK [126]. Specific mutations of DnaK designed to disrupt the crystallographic dimer interface and to probe its functional significance [127], displayed a defective chaperone activity and Hsp40 interactions. The Hsp70/Hsp40 chaperone system is also required to regulate client binding to Hsp90 and to load Hsp90 with a client protein [128]. The cochaperone Hop bridges the Hsp70 and Hsp90 chaperone systems [129], and inhibits the ATPase activity of Hsp90, stabilizing the client-loading conformation and facilitating client proteins handover [130].

In common with many chaperone systems, Hsp70/90-substrate interactions have proven challenging to study with traditional biophysical techniques due to their dynamic nature and compositional heterogeneity. This explains the requirement of alternative experimental methods to characterize them. A recent study combining crosslinking-mass spectrometry and structure modeling has demonstrated that phosphorylation of Thr504 on mammalian Hsp70 is critical for Hsp70 dimerization and promotes a client-loading complex comprising Hsp90, Hsp70, and Hsp40. Based on these results, the authors proposed a model in which Hsp70 antiparallel dimerization, stabilized by PTMs, positions the client for transfer from Hsp70 to Hsp90 [93]. The Hsp70 dimer interface is stabilized by electrostatic interactions, which are further strengthened by phosphorylation of Thr504. This phosphosite is located close to the hinge region between the two subdomains of the SBD, in a lysine-rich pocket that orients it towards the subunit interface for interactions with several lysine residues, which could stabilize the ADP conformation in the antiparallel dimer. A question that, however, remains unanswered is whether this Hsp70 antiparallel arrangement occurs during the functional cycle of the chaperones. In several pathologies, including cancer, the higher chaperone concentration and enhanced phosphorylation [131,132] could stimulate formation of antiparallel Hsp70 dimers, which in turn might facilitate substrate protein transfer from Hsp70 to Hsp90. Further work is necessary to find out if this phosphorylation is constitutive or is sensitive to the conditions that client proteins sense in the cellular context.

#### 5.3.4. Regulation of the Balance between Protein Folding and Degradation

Hsp70 usually interacts with client proteins several times before they fold properly. When these interaction cycles are not productive, it will target the client through different degradation pathways. A recently proposed model for Hsp70 functioning, puts forward that the chaperone provides a physical platform where client proteins, other chaperones and co-chaperones can bind. The fate of the client protein is governed by the set of protein interactions that are promoted under different cellular contexts [75]. During protein folding in eukarya, Hsp70 and Hsp90 chaperones work in a coordinate manner, Hsp90 acting downstream Hsp70. They are assisted by several cochaperones like Hsp40s, which have an essential role in targeting substrates to Hsp70, or Hsp70/Hsp90-organizing protein (HOP) that facilitates substrate transfer from Hsp70 to Hsp90 [130]. Protein degradation, in contrast, requires tagging of the protein with one or more ubiquitin molecules by the ubiquitin-proteasome system (UPS) to entry into the proteasome for degradation [133]. This connection between Hsp70 and the UPS is controlled by the interaction of one component of the UPS, the ubiquitin ligase CHIP with the Hsp70 C-terminus, which facilitates the ubiquitination of Hsp70-bound client proteins.

In proliferating cancer cells, phosphorylation of the C-terminus of Hsp70 (Thr636) and Hsp90 enhance their interaction with the co-chaperone HOP, increasing client protein stability and thus driving cancer growth. In contrast, nonphosphorylated chaperones preferentially bind CHIP, resulting in degradation of client proteins. Therefore, phosphorylation of the C-terminal substrate-binding domains of these chaperones regulates the client triaging process, by selecting the combination of proteins that interact with Hsp70 [94]. Interestingly, Thr636 phosphorylation has a small effect on the binding of other co-chaperones that share interacting surfaces on Hsp70 with CHIP and HOP [134]. Although the mechanism by which Hsp70 phosphorylation regulates the selectivity of cochaperone binding is far from being understood, it is clearly an interesting facet of the chaperone quality control.

#### 5.3.5. Regulation of the Cell Cycle Progression

Hsp70 phosphorylation has also been involved in the regulation of the cell cycle. The yeast Hsp70, Ssa1 is phosphorylated in Thr36 by different kinases, resulting in an important switch in Hsp70-client interactions that has been characterized by proteomic analysis of the Ssa1 interactome [96] (Figure 3C). In the G2/M phase, Clb cyclins activate Cdk1, which phosphorylates Ssa1, triggering displacement of the member of the Hsp40 family, Ydj1, and binding of the G1 cyclin Cln3 that is primed for degradation. Phosphorylation of Hsp70 Thr36 can also be achieved during nutrient limiting conditions by the stress CDK Pho85 activated by Pcl cyclins, which also drives exchange of Ydj1 by Cln3, which may be phosphorylated by Pho85 on the PEST domains. Both processes promote Cln3 degradation, preventing accumulation of Cln3 and resetting the cell for the next G1. The finding that CDK-dependent Thr38 phosphorylation on mammalian Hsc70 similarly regulates Cyclin D1 binding and activity, strongly suggests that Hsp70 chaperones can be dynamically activated to transduce cell signaling into cell cycle control.

#### 5.3.6. Hsp70 Phosphorylation Regulates Drug Resistance in Cancer Cells

Folates are key one-carbon donors in the process of DNA and RNA syntheses [135]. Methotrexate (MTX), a folate analog, is an antifolate chemotherapeutic agent that inhibits folate metabolism by inhibiting DHFR. Folates and folate analogs use different transport systems to enter cells. The reduced folate carrier (RFC), which displays high affinity for reduced folate and MTX, is the major route for the uptake of antifolate chemotherapeutic drugs in mammalian cells and tissues [136]. Aberrant functions of MTX transports can be the obstacle to successful transportation of MTX into cancer cells and may further lead to MTX resistance in cancer therapy. In this context, it has been shown that the NBD of Hsc70 binds MTX in different cancer cells [97]. Based on this experimental evidence, it was proposed that Hsc70 might mediate transport of MTX into the cell, cooperating with other MTX-interacting proteins. The finding that Hsc70 is phosphorylated on Tyr288 in MTX sensitive but not in resistant cells raised the proposal that Hsc70 phosphorylation can mediate entry of MTX into the cell and therefore modulate cancer growth and cellular resistance. As aforementioned, the identity of the kinases and phosphatases responsible for Tyr288 (de)phosphorylation remains unknown, and consequently, the possibility to modify its phosphorylation status for clinical purposes requires further studies.

#### 5.3.7. Regulation of Apoptosis

Hsp70 enhances cell growth, suppresses senescence, confers resistance to stress-induced apoptosis and serves as a good tumor marker [137,138]. A recent work has established that phosphorylation of Hsp70 at Ser486 was important for anti-apoptosis induced by serum starvation [98]. This PTM is mediated by the Retinoic Acid-Induced 16 (RAI16) protein, which after activation functions as a protein kinase A anchoring protein that also binds Hsp70. Thus, by holding in close proximity both PKA and Hsp70, RAI16 promotes Hsp70 phosphorylation, preventing cleavage of caspase-3 and apoptosis. Elevation of the cellular concentration of cAMP activates the PKA holoenzyme anchored to RAI16, which phosphorylates RAI16 on Ser325. This induces the recruitment of 14-3-3θ, which inhibits RAI16-mediated, PKA phosphorylation of Hsp70 and promotes apoptosis.

Together, these studies show that multiple kinases phosphorylate Hsp70 for selective signaling purposes, and reveal a complex phosphorylation-induced regulation of Hsp70 chaperone activity, which is essential to modulate diverse signaling pathways.

## 6. Phosphorylation as Part of the Chaperone Code

With the improvement of the experimental tools to identify PTMs, it has become clear that chaperones undergo extensive PTMs (Figure 2). However, the enzymes responsible for these modifications and the functional consequences that PTMs might have on these proteins remain largely unknown. Considering the overwhelming number of PTMs experimentally observed in chaperones, it has been proposed that a code, similar to that proposed for histones, does exist for chaperones [113]. Decrypting how this chaperone code regulates chaperone activity requires the detailed characterization of the effect that PTMs have on (i) the ATPase activity of Hsp70 and Hsp110; (ii) the conformation of the three components of this system; and (iii) the interaction of Hsp70 with its cochaperones and with a plethora of substrate proteins. It is of particular importance to note that, as far as phosphorylation is concerned, all chaperones and most likely some of their client proteins [139] can be phosphorylated at multiple sites, and therefore that the variable phosphorylation state of each of these components might be important to regulate their interaction and final functional outcome. Addressing the following questions is necessary to understand how the chaperone code might function.

### 6.1. How Phosphorylation Regulates the Conformation and Activity of These Chaperones?

Although most human proteins contain only few phosphorylation sites, some, as the chaperones studied in this work, do have multiple phosphorylation sites [140]. The ability of a dianionic phosphate group to establish extensive hydrogen bond networks and salt bridges with neighboring residues explains how phosphorylation affects stability, kinetics and dynamics [141]. Predicting the impact of phosphorylation on chaperone conformation and activity is not straightforward, mainly due to two reasons. First, the presence of multiple phosphorylation sites in the three chaperone families (Figure 2). Multisite phosphorylation complicates the accurate quantitative characterization of the phosphorylation status of the chaperones in the cell under different conditions, because multiple combinations of phosphorylated peptides can coexist, making their detection especially difficult. Without this identification, it is hard to assign a specific conformation to the distinct phosphorylation states that chaperones may have. Although mass spectrometry can sometimes provide insight into dynamics of post-translational modifications, a quantitative determination is not always possible [142].

Second, the flexible character of chaperone structure in solution poses an extra difficulty in assessing the effect of phosphorylation, and other PTMs, on the conformational cycle of chaperones [143]. Analyses of phosphorylation in different proteins revealed the diversity and heterogeneity of its effects on protein structure [144], as it can impact protein structure at local as well as global levels. Although recent crystallographic descriptions of the ATP-bound states gave the impression that different Hsp70s adopt the same domain-docked and domain-undocked states, growing experimental evidence suggests that in solution each chaperone has a complex conformational landscape and coexists as a heterogeneous ensemble of several conformations [145,146]. The population of each conformation, which in turn controls its ATPase and client interaction, seems to be under precise regulation by allosteric hotspots, regions that modulate chaperone conformational transitions. Subtle perturbations, such as amino acid substitutions, ligand binding or PTMs, at these allosteric hotspots have been shown to modify the chaperone conformational cycle, adjusting its chaperone activity [147]. As not all allosteric hotspots are fully conserved, functional diversity and real-time Hsp70 activity within the Hsp70 family might be regulated post-translationally, e.g., through covalent modifications, such as phosphorylation, and/or interactions with co-chaperones [51,148]. These studies also suggest that different members of the Hsp70 family apparently fine-tune their function post-translationally through adjustments of their conformational landscape rather than by altering chaperone structure [143]. Information on how phosphorylation could modulate the conformation of these chaperones is at present scarce.

Multiple phosphorylation can occur through different mechanisms. It can be sequential or random. In sequential phosphorylation, sites are modified in a strict order of events where phosphorylation of one site depends on the phosphorylation state of another. Sequential phosphorylation has been observed for several kinases, especially Ser/Thr kinases [149]. In contrast, random phosphorylation does not require a strict order of phosphorylation events. The identification of most kinases that phosphorylate these chaperones awaits further studies as does the mechanism they follow to phosphorylate multiple residues. Multisite phosphorylation can expand the regulation patterns, giving a more precise modulation of the phosphorylation-induced protein conformational change [150], and cooperatively regulating binding affinity to nucleotide, substrate proteins and co-chaperones [151,152]. Interestingly, large scale analyses revealed that multiple phosphorylation sites are not distributed randomly, often being clustered on a particular protein region [153,154]. Retinoblastoma protein (Rb) is an example of a protein with multiple phosphorylation sites and concerted phosphorylation patterns with very specific functional roles. Rb contains 13 different Ser/Thr phosphorylation sites that can be grouped into eight clusters, which mostly reside in flexible loop regions between structured regions or domains, and mediate domain-domain, domain-loop, and protein–protein interactions [155,156].

**Figure 4:**
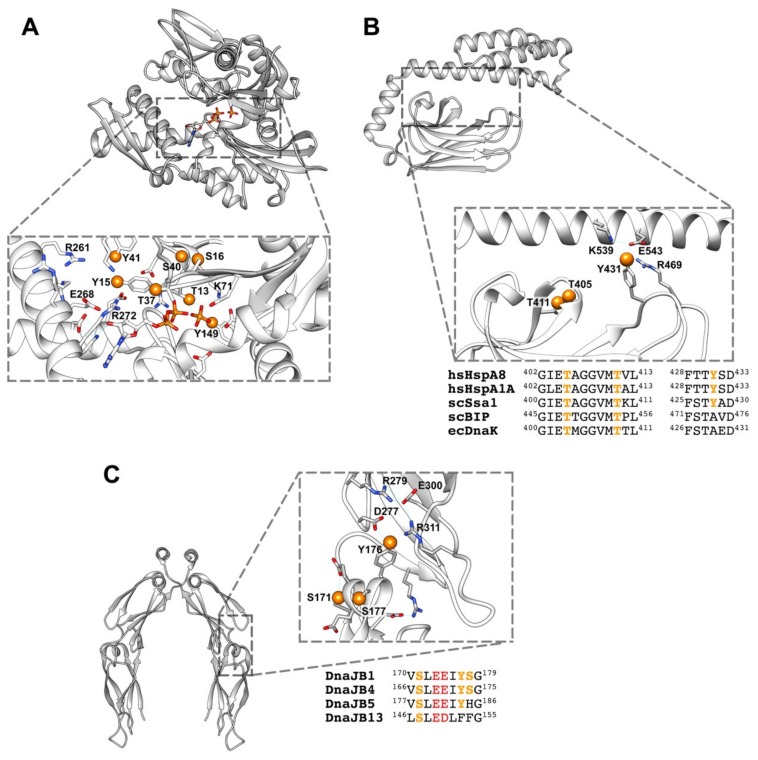
(**A**) Phosphorylated residues near the nucleotide binding site of Hsc70 (PDB ID: 4H5T). Orange spheres represent the oxygen of the hydroxyl groups to where the phosphate group would be attached. (**B**) The same for the substrate binding site of Hsc70 (PDB ID: 4PO2), showing the charged residues that could establish ionic interactions with the phosphate group. Alignment of different Hsp70s from human (hs), yeast (sc) and *E. coli* (ec). (**C**) Phosphorylated residues at the interface between the CTDI and CTDII subdomains of DnaJB1 (PDB ID: 2QLD). Alignment of distinct members of class B human Hsp40. Conserved acidic residues are shown in red and charged residues that could interact with the phosphate group are indicated as in (**B**). Alignments were done with ClustalW2. It is important to note that it is unknown whether the marked residues can be phosphorylated at the same time.

A similar scenario might occur in chaperones, as many of the experimentally observed, although not all, phosphorylated residues are close in the protein sequence or might form clusters upon folding, as seen in some of the available structures and models. As an example, the fully conserved residues Thr13, Tyr15, Ser16, Thr37, Tyr40, Ser41, and Tyr149 form one of these clusters in the NBD of Hsp70, surrounding the ATP binding pocket (Figure 4A). One of these residues (Thr13) interacts directly with the nucleotide and with Lys71, which is essential for ATP hydrolysis [157], and therefore, could modulate the ATPase activity of the chaperone. Two other amino acids, Tyr149 and Tyr15, might interfere with the orientation of the ligand either directly (Tyr149), or indirectly (Tyr15) through the interaction with residues Glu268 and Arg272 that participate in the correct positioning of the substrate [71]. Phosphorylation of loops L1,2 and L3,4 around the peptide binding site at the SBDβ subdomain of Hsp70 might also be important to regulate chaperone-client interaction (Figure 4B). A recent Molecular Dynamics (MD) study suggests that both loops establish contacts in the domain-undocked, substrate-bound conformation, whereas in the domain-docked conformation these interactions are disrupted [147]. The sensitivity of this conformational transition to the presence of the NBD and peptides indicates that they are part of the allosteric network that enables control of substrate binding and release. This suggestion has been experimentally proved by substituting different residues of these loops and, notably, by exchanging them in DnaK [158]. The mutated proteins showed a lower affinity for substrates [159], a change in the binding specificity for different substrates and in some cases the loss of the refolding activity of the chaperone [160,161]. Phosphorylation of Tyr431 in L3,4 and Thr405 and Thr411 in L1,2 might modulate their interaction as it could weaken inter-loop contacts, favoring the fully open arrangement of the loops (Figure 4B). In this conformation, the dynamics around the substrate binding loops would be essential for fast and efficient client binding to Hsp70. Phosphorylation of residues at these loops, as Tyr431, might also modify contacts on the SBDβ-SBDα interface that are important to regulate the allosteric properties of Hsp70 [162].

### 6.2. How Phosphorylation Modulates Intermolecular Interactions?

Protein–protein interactions control many cellular processes and main signaling pathways involve dense networks of interacting proteins and phosphorylation events. Analysis of phosphorylation sites on protein–protein binding interfaces has shown that protein interfaces of transient homo- and hetero-oligomers are statistically enriched in phosphorylation sites compared to non-interfacial protein surface sites [140,163]. There are different ways by which phosphorylation could modulate the chaperone activities that rely on protein–protein interactions, namely the holdase and foldase activities. Chaperones, as aforementioned, can inhibit aggregation of unstable protein conformations, and therefore display holdase activity. Phosphorylation of these proteins occurs in all protein domains, including those that have been related to client protein binding, namely the CTD, G/F and Zn-binding domain of Hsp40, and the SBDs of both Hsp70 and Hsp110 (see Figure 2). Furthermore, the fact that some substrates might also undergo phosphorylation [139,164], broadens the possibility that this PTM could regulate chaperone-substrate interactions. As an example of a possible effect of phosphorylation on a region of DnaJB1 involved in complex formation with substrate proteins, we analyze in Figure 4C the residues that have been found phosphorylated in the CTDI subdomain of DnaJB1 (Ser171, Tyr176, and Ser177). They form a cluster, together with the conserved Glu173 and Glu174 at the short helix located in the hinge that connects both CTD subdomains, and therefore their phosphorylation could modify the relative position of these protein regions. The phosphorylated residues could also interact with amino acids at the CTDII, such as the conserved Arg311, which is involved in a hydrogen bond network (Figure 4C). Moreover, we cannot rule out that the accumulation of five negatively charged residues on a seven amino acid long helix could destabilize it. Substitution of Tyr176 and Ser177 in members of the DnaJB class might also be related to their ability to bind distinct client proteins, and therefore to recruit Hsp70 for specific functions (Figure 4C).

Altering the affinity of the central Hsp70 chaperone for its cochaperones, i.e., in our case Hsp40 and Hsp110, is another way by which phosphorylation might affect the foldase and disaggregase activities of the system, as both require their cooperation. This change might be a direct consequence of introducing a charge group in the interaction surface or an indirect one, through an allosteric conformational change brought about by phosphorylation of (a) residue(s) not necessarily located at the protein–protein interface. This could be essential for the functional outcome, as cochaperones besides modulating the ATP cycle of their respective chaperone, direct it to specific cellular processes and client proteins. Thus, modifications that change the preference of the chaperones toward different cochaperones may ultimately have critical consequences on the fate of substrate proteins (folding or degradation), and on the processes in which the system is involved.

The large number of phosphorylation, and other PTMs, sites in the three proteins analyzed, scattered throughout all domains (Figure 2), suggests that synchronization of multiple PTMs through a combinatory code could time diverse chaperone functions and mediate recognition of multiple substrates with high specificity, as recently proposed for Hsp90 [165]. Phosphorylation can also modify other PTMS, a property known as crosstalk, as put forward for CSP (see above). Post-translational modification crosstalk occurs in those cases where phosphorylation of one or several residues influences the modification of another site(s). As an example, phosphorylation in some cases can modulate subsequent ubiquitylation and the crosstalk between phosphorylation and ubiquitylation is reciprocal, e.g., phosphorylation can be regulated by ubiquitylation and vice versa [166]. Therefore, unravelling the chaperone code will also require the characterization of the conformational and functional interplay between different PTMs. We are only starting to have a sense of the importance of PTMs in the regulation of Hsps and, clearly, there is a lot more to learn. Understanding the molecular mechanisms underlying this new and complex layer of chaperone regulation is a huge task that will need years of work.

## Figures and Tables

**Table 1:** Phosphorylation sites identified in members of the Hsp70, Hsp40, and Hsp110 chaperone families with known effects on their structure and function. hs: *Homo sapiens*; sc: *Saccharomyces cerevisiae*; ec: *Escherichia coli*; mm: *Mus musculus*.

Chaperone	Phosphorylation site(s)	Structural/Functional consequence(s)	Reference
hsDnaJB1	Ser149, Ser151 and Ser171	Inhibition of HSF1-mediated transcription	[86]
hsCSP (DnaJC5)	Ser10	Order-to-disorder transition. Modulation of neurotransmitter release by inhibiting binding to syntaxin and synaptotagmin	[87]
hsCSP (DnaJC5)	Ser10 and Ser34	Protection of the presynaptic terminal by promoting HSP70 chaperone activity	[88]
hsHsp105α (HSPH1)	Ser509	Inhibition of the Hsp105-induced suppression of Hsc70-mediated refolding	[89]
hsHsp70 (HSPA1A)	Ser631	Regulation of SOD2 import into the mitochondria and redox balance	[90]
hsHsp70 (HSPA1A)	Tyr524	Enhanced nuclear accumulation and heat-shock injury resistance	[91]
hsHsc70 (HSPA8)	Thr495	Inhibition of Hsp70 ATPase and refolding activities	[92]
ecHsp70 (DnaK)	Thr504	Stabilization of Hsp70 antiparallel dimers to position the client for transfer to Hsp90	[93]
hsHsc70 (HSPA8)	Thr636	Enhanced interaction with HOP	[94]
hsHsc70 (HSPA8)	Ser631 and Ser633	Recruitment of Hsc70 to the centrosomes leading to mitotic spindle elongation and prevention of apoptosis	[95]
scHsp70 (Ssa1)	Thr36	Regulation of the cell cycle progression	[96]
mmHsc70 (Hspa8)	Tyr288	Cell uptake of methotrexate	[97]
hsHsp70 (HSPA1A)	Ser486	Inhibition of apoptosis	[98]
hsHsp70 (HSPA1A)	Thr66	Promotion of K-fiber assembly and mitotic progression	[99]

## Abbreviations

PTM	Posttranslational modifications
Hsp	Heat shock protein
PQC	Protein quality control
NBD	Nucleotide binding domain
SBD	Substrate binding domain
CTD	C-terminal domain
TLA	Three letter acronym
ZBD	Zinc binding domain
Apg	ATP and peptide-binding protein in germ cells
EEVD	Glu-Glu-Val-Asp
sHsp	Small heat shock protein
αsyn	α-synuclein
Htt	Huntingtin
AS	Proline-rich acidic subdomain
NEF	Nucleotide exchange factor
TPR	Tetratricopeptide repeat
MK5	Mitogen-activated protein kinase-activated protein kinase 5
HSF1	Heat shock factor 1
CSP	Cysteine string protein
SNAP25	Synaptosomal nerve-associated protein 25
PKC	Protein kinase C
SOD2	Superoxide dismutase-2
CHIP	C-terminus of Hsc70 interacting protein
Akt1	RAC-alpha serine/threonine-protein kinase
UPS	Ubiquitin-proteasome system
PP2C	Protein phosphatase 2C
HOP	Hsp70/Hsp90 organizing protein
CDK	Cyclin-dependent protein kinase
G6PDH	Glucose 6 phosphate dehydrogenase
Hsc	Heat shock cognate
NMR	Nuclear magnetic resonance
